# Fibular length and vital capacity in the multinational Burden of Obstructive Lung Disease follow-up study

**DOI:** 10.1183/13993003.00690-2025

**Published:** 2025-07-24

**Authors:** Peter G.J. Burney, Tracey Nguyen, Dhiraj Agarwal, Christer Janson, Rune Nielsen, Padukudru Anand Mahesh, Rain Jögi, Gregory Erhabor, Meriam Denguezli, Karima El Rhazi, James Potts, Asaad A. Nafees, Parvaiz Koul, Stefanni N. Paraguas, André F.S. Amaral

**Affiliations:** 1 National Heart and Lung Institute, Imperial College, London, UK; 2 School of Medicine, Imperial College, London, UK; 3 Vadu Rural Health Program, KEM Hospital Research Centre, Pune, India; 4 Department of Clinical Science, University of Bergen, Bergen, Norway; 5 Department of Thoracic Medicine, Haukeland University Hospital, Bergen, Norway; 6 Department of Respiratory Medicine, JSS Medical College, JSSAHER, Mysore, India; 7 Lung Clinic, Tartu University Hospital, Tartu, Estonia; 8 Department of Medicine, Obafemi Awolowo University/Obafemi Awolowo University Teaching Hospital, Ile-Ife, Nigeria; 9 Université de Sousse, Faculté de Médecine de Sousse, Sousse, Tunisie; 10 Université de Monastir, Faculté de Médecine Dentaire de Monastir, Monastir, Tunisie; 11 Faculty of Medicine, Pharmacy and Dentistry, Research Laboratory of Epidemiology and Research in Health Sciences, Sidi Mohamed Ben Abdillah University, Hassan II University Hospital Centre, Fes, Morocco; 12 Department of Community Health Sciences, Aga Khan University, Karachi, Pakistan; 13 Sher-i-Kashmir Institute of Medical Sciences, Srinagar, India; 14 Philippine College of Chest Physicians, Quezon City, Philippines; 15 Philippine Heart Centre, Quezon City, Philippines; 16 NIHR Imperial Biomedical Research Centre, London, UK

## Abstract

The relative association of leg length and total height on lung volumes may be important for the clinical assessment of forced vital capacity (FVC) and for understanding critical periods in lung development. The expected size of the FVC in a healthy person generally takes account of their age, sex and size. Most commonly, size has been assessed as standing height or standing height squared. Louw *et al*. [1] suggested that sitting height gave a better assessment of lung function than standing height. Harik-Khan *et al*.

## Shareable abstract

**The association between long bone length and vital capacity was consistently small and non-significant in this multinational study** https://bit.ly/43FmQnH

*To the Editor:*

The relative association of leg length and total height on lung volumes may be important for the clinical assessment of forced vital capacity (FVC) and for understanding critical periods in lung development. The expected size of the FVC in a healthy person generally takes account of their age, sex and size. Most commonly, size has been assessed as standing height or standing height squared. Louw *et al*. [1] suggested that sitting height gave a better assessment of lung function than standing height. Harik-Khan *et al*. [2] suggested that differences in sitting height explained the difference in lung volumes in European Americans and African Americans, and suggested that this might be explained by the thoracic height being lower relative to total height in African Americans. Krause *et al*. [3], in a comparison of Danish and Inuit children, also suggested that a lower height-adjusted FVC in Danish children might be explained by shorter limb lengths in the Inuit children. Gunnell *et al*. [4] showed an association between leg length and both 1-s forced expiratory volume and FVC in the Scottish Midspan study.

In addition to an association between leg length and lung volumes, there has been an extensive investigation of the relationship of leg length to cardiovascular and metabolic health [5–7], conditions which are themselves associated with low lung volumes [8–11]. Furthermore, as leg length is particularly compromised by growth failure in infancy and childhood, it could indicate a mechanism that explains the association between short limb lengths, low lung volumes and cardiovascular and metabolic co-morbidities.

In the follow-up to the Burden of Obstructive Lung Disease (BOLD) study, we have investigated the relationship between fibular length and spirometry values of lung volume.

A detailed description of the BOLD follow-up study has been reported elsewhere [12]. Briefly, study sites were selected if they had participated in the BOLD baseline survey, were able to undertake a follow-up survey and were either in low- or middle-income or the Nordic countries. All those who had provided good quality lung function measurements at baseline were eligible for follow-up. Those who were available and consented were administered questionnaires, had their lung function tested and were measured by trained fieldworkers according to a common protocol. Standing height was measured using a stadiometer to the nearest centimetre. Fibular length was measured with a flexible tape from the head of the fibula, at the knee, to the lateral malleolus, at the ankle. Waist circumference was measured at the end of normal expiration midway between the iliac crest and the costal margin. For each of these assessments, two measurements were made to the nearest millimetre and the average of the two used as the estimate. Lung function was assessed using the ndd EasyOne spirometer (ndd Medizintechnik AG, Zurich, Switzerland) before and after inhaling 200 μg salbutamol (albuterol) through a spacer (Able, Clement-Clark International).

Data were recorded as percentages or as mean±sd. Inverse probability weights were used to account for missingness in the follow-up sample. For each sex separately, FVC was regressed against age, height squared, the ratio of fibular length to standing height, waist circumference and smoking history using meta-analytic techniques to allow for the clustered nature of the study by region and site. The analysis was cross-sectional and undertaken using Stata v.17.

The protocol was approved by the Imperial College London Research Ethics Committee (ref. 17IC4272) and by the relevant ethics committee in each study site.

A total of 2440 men and 3060 women had complete data. The mean age of the participants ranged from 54 years (Mysore, India) to 69 years (Uppsala, Sweden) in men, and from 53 years (Mysore, India) to 69 years (Tartu, Estonia) in women. Mean height ranged from 162 cm (Nampicuan and Talagtug, Philippines) to 179 cm (Reykjavik, Iceland) in men, and from 151 cm (Pune, India) to 165 cm (Reykjavik, Iceland) in women. Mean fibular length ranged from 37 cm (Naryn, Kyrgyzstan) to 47 cm (Jamaica) in men, and from 34 cm (Mysore, India) to 43 cm (Jamaica) in women. Mean waist circumference ranged from 78 cm (Chikwawa, Malawi) to 106 cm (Tartu, Estonia) in men, and from 79 cm (Pune, India) to 106 cm (Sousse, Tunisia) in women. Among men, only 34% had ever smoked and half had given up. Among women, only 9% had ever smoked and only 5% were still smoking. Mean FVC ranged from 2.91 L (Pune, India) to 4.39 L (Reykjavik, Iceland) in men, and from 1.95 L (Pune, India) to 3.09 L (Reykjavik, Iceland) in women.

A greater post-bronchodilator FVC was associated with greater height (m^2^), younger age and lower waist circumference (table 1). The association with smoking was only significant for ex-smoking in men. The beta-coefficient for the ratio of fibula length to height was not significant in either women (β=0.561, 95% CI −0.748 to 1.869) or men (β=1.796, 95% CI −0.335 to 3.928), and the beta-coefficients did not vary significantly between BOLD sites in either sex.

**TABLE 1:** Association of forced vital capacity with anthropometric measurements and smoking status in the Burden of Obstructive Lung Disease follow-up study

	Women (n=3060)				Men (n=2440)			
	β	95% CI	I^2^	p-value (heterogeneity)^#^	β	95% CI	I^2^	p-value (heterogeneity)^#^
**Age (per year)**	**−0.020**	**−0.023, −0.016**	57	0.003	**−0.022**	**−0.028, −0.016**	70	<0.001
**Current smoker**	0.099	−0.111, 0.308	64	0.003	−0.102	−0.228, 0.023	47	0.020
**Ex-smoker**	0.086	−0.027, 0.199	45	0.055	**−0.084**	**−0.165, −0.003**	20	0.22
**Height-squared (m^2^)**	**1.066**	**0.863, 1.269**	74	<0.001	**1.393**	**1.188, 1.597**	55	0.005
**Fibula/height**	0.561	−0.748, 1.869	29	0.14	1.796	−0.335, 3.928	36	0.073
**Waist**	**−0.005**	**−0.007, −0.003**	35	0.089	**−0.011**	**−0.014, −0.007**	56	0.003

Note: any smoking history is rare among women, and current smoking is rare among men. ^#^: probability that I^2^ is not significant. Bold typeface indicates significant beta-coefficient (p<0.05).

In this multinational study, we have not found a clear association between the ratio of fibular length to total height and FVC. The associations that we have found, though positive and consistent across sites, are small and not significant. We do not exclude a positive association, but any association is likely to be small. After standing height, the next most important dimension associated with FVC was the waist circumference, possibly through central obesity restricting movement of the diaphragm.

We chose to measure fibular length rather than leg length or sitting height, first because of ease of measurement and standardisation, and secondly because secular changes in growth have been greater in lower than in upper limbs, and greater in the lower leg than in the upper leg [13]. As with all other measurements in the study, training was standardised for all sites. The lung function was also standardised by training to a common protocol and by central over-reading of all measurements, and re-training of technicians whenever quality began to deteriorate.

The BOLD study includes a diverse population spread over a wide geographical area. The variation in fibular length is also wide, the largest average measurements being found in sub-Saharan Africa and Jamaica. The larger mean values in sub-Saharan Africa and Jamaica would seem to be at odds with the general hypothesis that fibular length is predominantly determined by nutrition in childhood and adolescence, and that leg measurements of all sorts are associated with better environments, nutrition and health [14]. There has always been an element of paradox in the hypothesis that longer relative leg length in the more deprived African American population might explain their low FVC relative to standing height. The lack of any significant heterogeneity in this result suggests that fibula-to-height ratio is not an explanation of FVC in any specific site or cluster of sites.

There is evidence for regional differences in the relationship of FVC to age and height [15], but in this large multinational study we were not able to demonstrate any substantive association between fibular length and FVC.

## Shareable PDF

10.1183/13993003.00690-2025.Shareable1 This PDF extract can be shared freely online.
Shareable PDF

ERJ-00690-2025.Shareable
