# Supplementary material for: Fibular length and vital capacity in the multinational Burden of Obstructive Lung Disease follow-up study
Source: Eur Respir J. 2025 Jul 24;66(1):2500690. doi: 10.1183/13993003.00690-2025 (PMC12287606; doi:10.1183/13993003.00690-2025)
Supplement: Supplementary file 1 [file ERJ-00690-2025.Shareable.pdf]

EUROPEAN RESPIRATORY JOURNAL
RESEARCH LETTER
P.G.J. BURNEY ET AL.

Fibular length and vital capacity in the multinational Burden of Obstructive Lung Disease follow-up study

Peter G.J. Burney[1], Tracey Nguyen[2], Dhiraj Agarwal[3], Christer Janson[4], Rune Nielsen[4,5], Padukudru Anand Mahesh[6], Rain Jögi[7], Gregory Erhabor[8], Meriam Denguezli[9,10], Karima El Rhazi[11], James Potts[1], Asaad A. Nafees[12], Parvaiz Koul[13], Stefanni N. Paraguas[14,15] and André F.S. Amaral[1,16] for the Burden of Obstructive Lung Disease collaborators

[1]National Heart and Lung Institute, Imperial College, London, UK. [2]School of Medicine, Imperial College, London, UK. [3]Vadu Rural Health Program, KEM Hospital Research Centre, Pune, India. [4]Department of Clinical Science, University of Bergen, Bergen, Norway. [5]Department of Thoracic Medicine, Haukeland University Hospital, Bergen, Norway. [6]Department of Respiratory Medicine, JSS Medical College, JSSAHER, Mysore, India. [7]Lung Clinic, Tartu University Hospital, Tartu, Estonia. [8]Department of Medicine, Obafemi Awolowo University/Obafemi Awolowo University Teaching Hospital, Ile-Ife, Nigeria. [9]Université de Sousse, Faculté de Médecine de Sousse, Sousse, Tunisie. [10]Université de Monastir, Faculté de Médecine Dentaire de Monastir, Monastir, Tunisie. [11]Faculty of Medicine, Pharmacy and Dentistry, Research Laboratory of Epidemiology and Research in Health Sciences, Sidi Mohamed Ben Abdillah University, Hassan II University Hospital Centre, Fes, Morocco. [12]Department of Community Health Sciences, Aga Khan University, Karachi, Pakistan. [13]Sher-i-Kashmir Institute of Medical Sciences, Srinagar, India. [14]Philippine College of Chest Physicians, Quezon City, Philippines. [15]Philippine Heart Centre, Quezon City, Philippines. [16]NIHR Imperial Biomedical Research Centre, London, UK.

Corresponding author: Peter G.J. Burney (p.burney@imperial.ac.uk)

Shareable abstract (@ERSpublications)
The association between long bone length and vital capacity was consistently small and non-significant in this multinational study https://bit.ly/43FmQnH

Cite this article as: Burney PGJ, Nguyen T, Agarwal D, *et al*. Fibular length and vital capacity in the multinational Burden of Obstructive Lung Disease follow-up study. *Eur Respir J* 2025; 66: 2500690 [DOI: 10.1183/13993003.00690-2025].

This PDF extract can be shared freely online.

Copyright ©The authors 2025

This version is distributed under the terms of the Creative Commons Attribution Licence 4.0.

Received: 1 April 2025
Accepted: 7 June 2025

To the Editor:

The relative association of leg length and total height on lung volumes may be important for the clinical assessment of forced vital capacity (FVC) and for understanding critical periods in lung development. The expected size of the FVC in a healthy person generally takes account of their age, sex and size. Most commonly, size has been assessed as standing height or standing height squared. Louw *et al*. [1] suggested that sitting height gave a better assessment of lung function than standing height. Harik-Khan *et al*. [2] suggested that differences in sitting height explained the difference in lung volumes in European Americans and African Americans, and suggested that this might be explained by the thoracic height being lower relative to total height in African Americans. Krause *et al*. [3], in a comparison of Danish and Inuit children, also suggested that a lower height-adjusted FVC in Danish children might be explained by shorter limb lengths in the Inuit children. Gunnell *et al*. [4] showed an association between leg length and both 1-s forced expiratory volume and FVC in the Scottish Midspan study.
